# Cardiac structure and function in schizophrenia: cardiac magnetic resonance imaging study

**DOI:** 10.1192/bjp.2019.268

**Published:** 2020-08

**Authors:** Emanuele F. Osimo, Stefan P. Brugger, Antonio de Marvao, Toby Pillinger, Thomas Whitehurst, Ben Statton, Marina Quinlan, Alaine Berry, Stuart A. Cook, Declan P. O'Regan, Oliver D. Howes

**Affiliations:** 1 Academic Clinical Fellow in Psychiatry, MRC London Institute of Medical Sciences, Faculty of Medicine, Imperial College London, Hammersmith Hospital Campus; and Department of Psychiatry, University of Cambridge; and Cambridgeshire and Peterborough NHS Foundation Trust, Cambridge, UK; 2 Academic Clinical Fellow in Psychiatry, MRC London Institute of Medical Sciences, Faculty of Medicine, Imperial College London, Hammersmith Hospital Campus, UK; 3 Clinical Lecturer in Cardiology, MRC London Institute of Medical Sciences, Faculty of Medicine, Imperial College London, Hammersmith Hospital Campus, UK; 4 Academic Clinical Fellow in Psychiatry, MRC London Institute of Medical Sciences, Faculty of Medicine, Imperial College London, Hammersmith Hospital Campus; and Department of Psychosis Studies, Institute of Psychiatry, Psychology and Neuroscience, King's College London, UK; 5 Clinical Research Fellow, MRC London Institute of Medical Sciences, Faculty of Medicine, Imperial College London, Hammersmith Hospital Campus, UK; 6 Lead MR Radiographer, MRC London Institute of Medical Sciences, Faculty of Medicine, Imperial College London, Hammersmith Hospital Campus, UK; 7 MR Radiographer, MRC London Institute of Medical Sciences, Faculty of Medicine, Imperial College London, Hammersmith Hospital Campus, UK; 8 Professor of Clinical and Molecular Cardiology, MRC London Institute of Medical Sciences, Faculty of Medicine, Imperial College London, Hammersmith Hospital Campus, UK; 9 Reader in Imaging Sciences (Consultant Radiologist), MRC London Institute of Medical Sciences, Faculty of Medicine, Imperial College London, Hammersmith Hospital Campus, UK; 10 Professor of Molecular Psychiatry, MRC London Institute of Medical Sciences, Faculty of Medicine, Imperial College London, Hammersmith Hospital Campus; and Institute of Psychiatry, Psychology and Neuroscience, King's College London, UK

**Keywords:** Schizophrenia, cardiac, function, structure, remodelling, cardiovascular, risk

## Abstract

**Background:**

Heart disease is the leading cause of death in schizophrenia. However, there has been little research directly examining cardiac function in schizophrenia.

**Aims:**

To investigate cardiac structure and function in individuals with schizophrenia using cardiac magnetic resonance imaging (CMR) after excluding medical and metabolic comorbidity.

**Method:**

In total, 80 participants underwent CMR to determine biventricular volumes and function and measures of blood pressure, physical activity and glycated haemoglobin levels. Individuals with schizophrenia (‘patients’) and controls were matched for age, gender, ethnicity and body surface area.

**Results:**

Patients had significantly smaller indexed left ventricular (LV) end-diastolic volume (effect size *d* = −0.82, *P* = 0.001), LV end-systolic volume (*d* = −0.58, *P* = 0.02), LV stroke volume (*d* = −0.85, *P* = 0.001), right ventricular (RV) end-diastolic volume (*d* = −0.79, *P* = 0.002), RV end-systolic volume (*d* = −0.58, *P* = 0.02), and RV stroke volume (*d* = −0.87, *P* = 0.001) but unaltered ejection fractions relative to controls. LV concentricity (*d* = 0.73, *P* = 0.003) and septal thickness (*d* = 1.13, *P* < 0.001) were significantly larger in the patients. Mean concentricity in patients was above the reference range. The findings were largely unchanged after adjusting for smoking and/or exercise levels and were independent of medication dose and duration.

**Conclusions:**

Individuals with schizophrenia show evidence of concentric cardiac remodelling compared with healthy controls of a similar age, gender, ethnicity, body surface area and blood pressure, and independent of smoking and activity levels. This could be contributing to the excess cardiovascular mortality observed in schizophrenia. Future studies should investigate the contribution of antipsychotic medication to these changes.

Schizophrenia is a major mental illness, affecting approximately 0.4–0.7% of the world population,^1^ equivalent to 30–52.5 million people. People with schizophrenia have a two- to threefold greater mortality compared with the general population,^2^ and their life expectancy is 10–25 years shorter.^3^ In addition to the higher suicide risk, mortality due to natural causes has been estimated to be up to eight times higher than expected.^2^ Despite reductions in mortality in the general population over the past 30 years (both all-cause and cardiovascular),^4^ the mortality gap between people with schizophrenia and the healthy population is not reducing, and may even be increasing.^5^ Cardiovascular disease (CVD) accounts for a large proportion of the loss of life in schizophrenia,^6^ accounting for 14.3 life-years lost^7^ or 60% of deaths,^8^ on average, and its importance as a cause of death has been increasing in both US-based^8^ and northern European^4^ populations. Moreover, people with schizophrenia are less likely to receive investigation and treatment for cardiometabolic comorbidity than the general population,^9^ which may contribute to their excess cardiac mortality.

Despite this wealth of epidemiological evidence that associates schizophrenia with cardiovascular disease and premature death, few studies have directly investigated cardiac function in people with schizophrenia. These have generally shown reductions in left ventricular ejection fraction and increases in left ventricular mass in schizophrenia.^10–12^ However, interpretation of these findings is complicated, as the studies did not consistently control for known determinants of cardiovascular structure and function, including body mass index (BMI), gender and comorbid metabolic conditions, which are factors that could independently account for the changes that were found. Consequently, it remains unknown whether schizophrenia is associated with cardiac structural or functional alterations, or whether potential confounds, such as medical and metabolic comorbidity explain the differences reported. Furthermore, previous studies have used transthoracic echocardiography to assess cardiac function, which provides less accurate and less reproducible volumetric and functional assessment of the heart compared with cardiac magnetic resonance imaging (CMR).^13^

In view of this, we aimed to determine whether cardiac structure and function is altered in people with schizophrenia using CMR, the gold standard *in vivo* measure of cardiac structural and functional assessment.^14^ Using a cohort of people with schizophrenia free from metabolic or medical comorbidities and healthy controls matched for known determinants of cardiovascular structure and function, including age, gender, ethnicity and body surface area (BSA), we tested the hypothesis that people with schizophrenia show heart changes that could account for the increased cardiovascular mortality independent of established cardiovascular risk factors such as medical and metabolic comorbidity.

## Method

### Participants

A total of 40 people with schizophrenia (‘patients’, mean age 39.8 years, s.d. = 9.6 years) were recruited from South London and the Maudsley NHS Foundation Trust and from Central and North West London NHS Foundation Trust in London, UK. A further 40 matched healthy controls (mean age 38.9 years, s.d. = 9.4 years) were recruited after matching for age (±3 years), ethnicity, gender and body surface area (BSA, ±2; BSA is a parameter similar to body mass index, BMI) through the Hammersmith Hospital Healthy Volunteer Panel in London and from the UK Digital Heart Project at Imperial College London (volunteers recruited via advertisement as previously described^15^). Recruitment took place between September 2015 and December 2018.

Exclusion criteria for all participants were: age <18 or >65 years; pregnancy or breastfeeding; a history of cardiometabolic disease, including diabetes, hypertension, dyslipidaemia, ischaemic heart disease, any vascular disorder, other history of congenital/structural cardiac disease; and history of significant or continuing substance misuse. The inclusion criterion for patients was an ICD-10 diagnosis of schizophrenia. Exclusion criterion for healthy controls was a personal history or first-degree family history of schizophrenia or other psychotic disorder.

Written informed consent was obtained from all volunteers. The authors assert that all procedures contributing to this work comply with the ethical standards of the relevant national and institutional committees on human experimentation and with the Helsinki Declaration of 1975, as revised in 2008. All procedures involving human participants were approved by the London – Camberwell St Giles research ethics committee.

### Assessment of participants

Physical assessment, study questionnaires, and CMR were all performed during the same study visit. All patients were assessed at the time of CMR imaging using the Positive and Negative Syndrome Scale (PANSS) for Schizophrenia^16^ by a study psychiatrist. A blood sample for HbA_1c_ was collected from a subsample of 38 matched participants (19 patients and 19 controls). Participants were seated in a quiet, temperature-controlled waiting-room and given time to relax. A study doctor then performed a screening interview, clinical examination and supervised the CMR scan. The presence of medical comorbidities was assessed directly by interviewing the participants, by examining the list of active prescriptions and, where in doubt, by examining general practice records. Brachial blood pressure measurement was performed following 5 min rest using a validated oscillometric device (Omron M7, Omron Corporation, Kyoto, Japan). The first of three measures was discarded, and the second two values were averaged to provide the final reading. Physical activity grading was based on the Copenhagen City Heart Study Leisure Time Physical Activity Questionnaire, performed on the same day as the CMR. Categories of activity were based on participants' level of activity over the preceding 12 months, ranging from level 1 (almost entirely sedentary) to level 4 (>5 hours of exercise per week).^17^ Plasma HbA_1c_ was analysed using the Tosoh G8 HPLC Analyzer (Tosoh Bioscience, San Francisco, USA). Antipsychotic doses were converted into chlorpromazine equivalents as described by Andreasen and colleagues.^18^

### Magnetic resonance imaging protocol

**Fig. 1:**
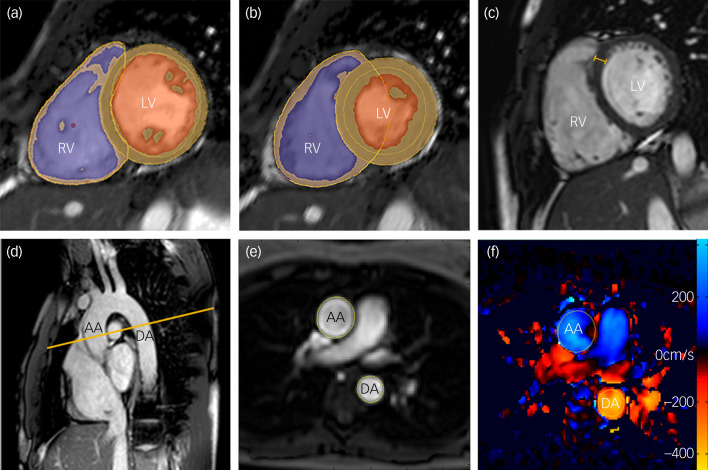
Magnetic resonance images demonstrating the assessment of biventricular volumes and function in an adult participant.

The CMR was performed at a single site for all 80 participants. Owing to a hardware upgrade the first 21 patients and matched controls were scanned on a 1.5 T Philips Achieva (Best, The Netherlands) using a 32-channel cardiac coil and the remaining 19 patients and matched controls were scanned on a 3 T Siemens Magnetom Prisma (Erlangen, Germany) using a combination of the 18-channel body coil and 12 elements of the 32-channel spine coil. Matched patients and controls were scanned on the same scanner. All images used a vectorcardiographic technique for electrocardiogram (ECG) synchronisation and were acquired during breath-hold at expiration. A standard clinical protocol for assessing biventricular function and volumes was followed according to published international guidelines.^19^ Participants whose images were degraded by respiration or ECG synchronisation artefacts to such an extent that cardiac contours could not be clearly identified were excluded from the analysis. Images were stored on an open-source database.^20^ Volumetric analysis of the cine images was performed using CMRtools (Cardiovascular Imaging Solutions, London, UK) for Windows by experienced operators masked to the diagnosis. The epicardial and endocardial borders were manually contoured at end-diastole and end-systole in each of the short-axis slices. Semi-automated, signal intensity-based thresholding was then used to identify the papillary muscles; these were included in the left ventricular mass and excluded from volumetric measurements. To increase the accuracy of the analysis, the valve positions at end-diastole and end-systole were manually identified on the three long-axis images, allowing the valve planes to be tracked through the cardiac cycle (Fig. 1). Volumes and mass were indexed to body surface area calculated using the Mosteller formula: BSA = √(height [cm] × weight [kg]/3600). Indexed volumetric data were left ventricular mass (LVMi), left ventricular and right ventricular end-diastolic volumes (LVEDVi and RVEDVi), left ventricular and right ventricular end-systolic volumes (LVESVi and RVESVi), and left ventricular and right ventricular stroke volumes (LVSVi and RVSVi). The end-diastolic volume (EDV) and end-systolic volume (ESV) were calculated using Simpson's method.^21^ Stroke volume (SV) was calculated as EDV − ESV and ejection fraction as SV/EDV × 100. Left ventricular mass was the total epicardial volume minus the total endocardial volume multiplied by the specific density of the myocardium (1.05 g/mL). Maximal end-diastolic wall thickness was measured through the basal anteroseptal wall using a short-axis CMR image (Fig. 1(c)). Left ventricular concentricity was calculated as the ratio LVM/LVEDV. Pulse-wave velocity was calculated using the method described by Corden *et al*.^22^ (Fig. 1(d–f)).

**(a), (b) Ventricular function and mass.** Endo- and epicardial contours of the left and right ventricle in diastole (a) and systole (b). Myocardium segmentation shown by orange lines, left ventricular (LV) cavity shown in orange and right ventricular (RV) cavity shown in purple. For ventricular function assessment, the endocardial and epicardial contours of the left and right ventricle were delineated in diastole (a) and systole (b) on a stack of short-axis slices covering the entire ventricles.**(c) Septal thickness measurement shown by the orange calliper.(d)–(f) Pulse-wave velocity.** Transverse cardiac magnetic resonance imaging slice through the aortic arch (d) displaying the magnitude- (e) and velocity- (f) encoded images through the aortic arch. Yellow contours outline the ascending aorta (AA) and descending aorta (DA). Blood flow through the aorta is encoded with a signal intensity relative to its velocity (cm/s), shown here on a blue/red/yellow colour scale (f).

### Statistical analysis

Differences among patients and controls were tested using χ^2^-tests for categorical variables, Wilcoxon rank sum tests for non-normally distributed values and analysis of variance (ANOVA) for normally distributed measures. Effect sizes were calculated using Cohen's *d* measure.

As there were significant group differences in smoking and physical activity levels, sensitivity analyses were conducted using generalised linear models with smoking, physical activity levels and scanner (1.5 T or 3 T) as covariates, the cardiac measures as the dependent variables and group as the independent variable. Both smoking and physical activity were used as continuous variables (not binarised, but as number of cigarettes and questionnaire scores), because (a) there is a dose–response relationship between smoking and CVD^23^ and between physical activity levels and CVD;^24^ (b) both the number of cigarettes and the physical activity scale were approximately linear (with the scale homogeneously measuring the number of activity hours per week, e.g. 0–2, 2–4, >4); and (c) categorisation of continuously distributed exposure variables requires a step-function of risk that assumes homogeneity of risk within groups, leading to both a loss of power and inaccurate estimation.^25^

To explore the possibility of a dose–response relationship between medication and cardiac phenotypes, linear regression was used to correlate cardiac measures with the natural logarithm of total chlorpromazine equivalents (to normalise the data distribution). The overall proportion of variance explained by linear models was calculated using *R*^2^ measures.

*P*-values were adjusted using the Benjamini–Hochberg method, and results were considered significant when the false-discovery rate (*q*) was <0.05. Statistical analyses and graph plotting were performed in R (R Foundation for Statistical Computing, 2019, Vienna, Austria) for Mac Os 10.13.

## Results

A total of 80 participants were recruited for this study. One healthy control had to be excluded because of poor image quality on CMR. Participants' characteristics are described in Table 1. At the time of assessment, all patients were being treated with antipsychotics (clozapine, *n* = 28; olanzapine, *n* = 10; and risperidone, *n* = 2). There were no significant group differences for age, gender, ethnicity, BSA, HbA_1c_, systolic and diastolic blood pressure. However, smoking levels and weekly physical activity levels were significantly different between groups.

**Table 1:** Sample characteristics

Characteristic	Schizophrenia group	Healthy controls	Test statistic and *P*
Sample size	40	39	
Male gender, *n* (%)	31 (77.5%)	32 (82.0%)	*χ*^2^ = 0.2; d.f. = 1; *P* = 0.61
White ethnicity, *n* (%)	19 (47.5%)	21 (53.9%)	*χ*^2^ = 0.32; d.f. = 1; *P* = 0.57
Age, years: mean (s.d.)	39.80 (9.55)	38.92 (9.38)	*F* = 0.17, d.f. = 1, *P* = 0.68
Number of cigarettes smoked per day, median (IQR; minimum; maximum)	1 (4.25; 0; 35)	0 (0; 0; 10)	Wilcoxon rank sum test *P* <0.001
Proportion of smokers	60%	15.4%	
Activity score, median (IQR)	2 (0)	3 (1.5)	Wilcoxon rank sum test *P* <0.001
Proportion with sedentary behaviour (score of 1 on 4-point scale)	17.50%	12.82%	
BSA, m^2^: mean (s.d.)	2.05 (0.27)	1.95 (0.22)	*F* = 3.45, d.f. = 1, *P* = 0.07
Systolic BP, mmHg: mean (s.d.)	124.64 (11.11)	122.05 (12.45)	*F* = 0.90, d.f. = 1, *P* = 0.35
Diastolic BP, mmHg: mean (s.d.)	81.22 (9.50)	77.78 (9.58)	*F* = 2.44, d.f. = 1, *P* = 0.12
HbA_1c_, mmol/mol: mean (s.d.)^a^	36.28 (6.02)	33.90 (7.92)	*F* = 1.04, d.f. = 1, *P* = 0.31
Chlorpromazine equivalent dose, mg/day: median (IQR)	377 (290)	0 (NA)	NA
Duration of treatment, years: median (IQR)	12 (13)	NA	NA
Total PANSS score, median (IQR)	62 (35.5)	NA	NA

IQR, interquartile range; BSA, body surface area (calculated using Mosteller formula); BP, blood pressure; HbA_1c_, glycated haemoglobin; NA, not applicable.

a. A blood sample for HbA_1c_ was collected from a subsample of 38 matched participants (19 patients and 19 controls).

### Cardiac measurements of ventricular structure and function in patients and matched healthy controls

**Fig. 2:**
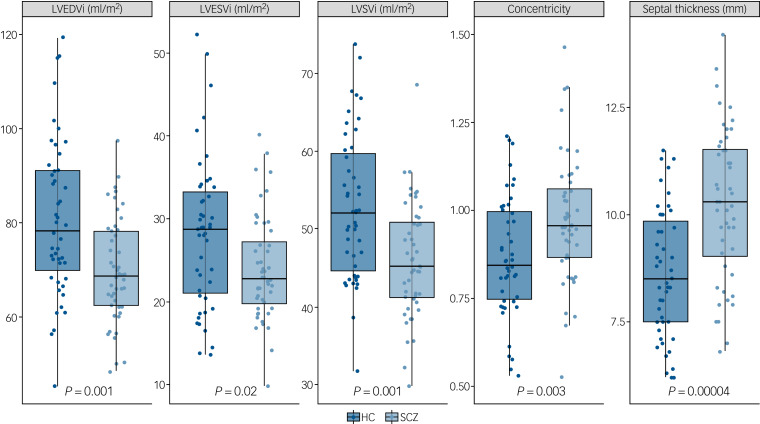
Left ventricular cardiac measurements in patients with schizophrenia (SCZ) and healthy controls (HC).

**Fig. 3:**
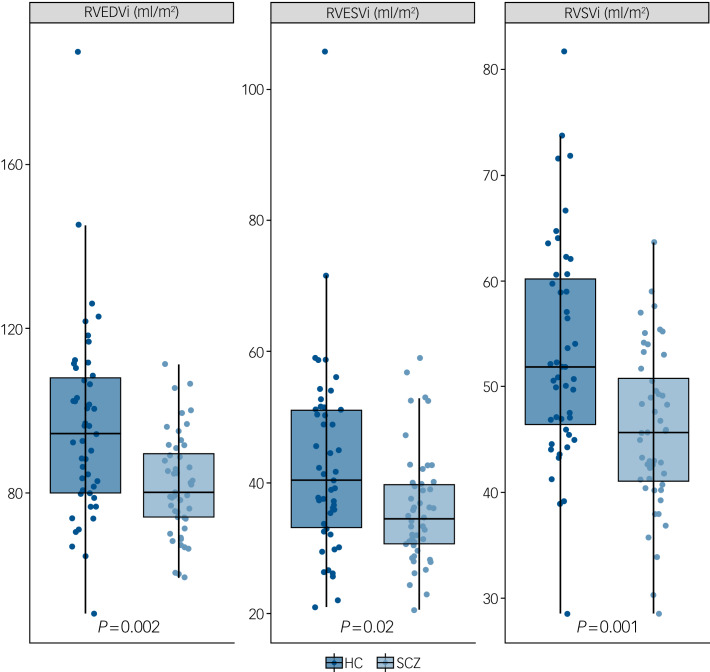
Right ventricular cardiac measurements in patients with schizophrenia (SCZ) and healthy controls (HC).

Supplementary Table 1 (available at https://doi.org/10.1192/bjp.2019.268) and Figs 2 and 3 describe CMR-derived measures of cardiac volume and function according to diagnostic status. After adjusting results for multiple testing, patients had significantly smaller LVEDVi (*d* = −0.82, *F* = −11.90, *P* = 0.001), LVESVi (*d* = −0.58, *F* = −4.65, *P* = 0.02), left ventricular stroke volume (*d* = −0.85, *F* = −7.25, *P* = 0.001) (Fig. 2), RVEDVi (*d* = −0.79, *F* = −15.32, *P* = 0.002), RVESVi (*d* = −0.58, *F* = −7.36, *P* = 0.02) and right ventricular stroke volume (*d* = −0.87, *F* = −8.09, *P* = 0.001), with large effect sizes (Fig. 3). Left ventricular concentricity (*d* = 0.73, *F* = 0.13, *P* = 0.003) and septal thickness (*d* = 1.13, *F* = 1.71, *P* <0.001) were significantly larger in the patients than in the matched healthy controls, with large to very large effect sizes (Fig. 2). Mean concentricity in patients was above reference ranges for male adult populations, with 77.5% of patients and 41% of controls above the threshold (*χ*^2^ = 9.4, d.f. = 1, *P* = 0.002). The means of all other parameters were within reference ranges in both patients and controls (supplementary Table 1). There were no significant differences between groups in left ventricular mass, biventricular ejection fractions or pulse-wave velocity (supplementary Table 1 and Fig. 1).

Graphs show individual values and box and whisker plots (the solid horizontal line is the median, the lower and upper hinges correspond to the first and third quartiles (the 25th and 75th percentiles), and the whiskers extend from the hinge to the largest/smallest value no further than 1.5 times the interquartile range from the hinge). *P*-values are adjusted for multiple testing. Patients had significantly smaller indexed left ventricular end-diastolic volume (LVEDVi), indexed left ventricular end-systolic volume (LVESVi) and indexed left ventricular stroke volume (LVSVi). Left ventricular concentricity and septal thickness were significantly larger in patients compared with matched healthy controls.

Graphs show individual values and box and whisker plots (the solid horizontal line is the median, the lower and upper hinges correspond to the first and third quartiles (the 25th and 75th percentiles), and the whiskers extends from the hinge to the largest/smallest value no further than 1.5 times the interquartile range from the hinge). *P*-values are adjusted for multiple testing. Patients had significantly smaller indexed right ventricular end-diastolic volume (RVEDVi), indexed right ventricular end-systolic volume (RVESVi) and indexed right ventricular stroke volume (RVSVi) compared with matched healthy controls.

#### Sensitivity analyses

In sensitivity analyses, we explored whether significant differences between patients and controls in terms of cigarettes smoked per day and number of hours of weekly exercise contributed to the differences in cardiac measurements we observed. Supplementary Table 1 shows that, after adjusting the model for the number of cigarettes smoked per day, patients still exhibited significantly smaller LVEDVi (*P* = 0.004), LVESVi (*P* = 0.04), LVSVi (*P* = 0.003), RVEDVi (*P* = 0.006), RVESVi (*P* = 0.04) and RVSVi (*P* = 0.003), whereas left ventricular concentricity (*P* = 0.02) and septal thickness (*P* < 0.001) were both still significantly larger. Significant results were essentially the same if analyses were adjusted for smoking binarised as smoking/not smoking instead (supplementary Table 2). After covarying for physical activity levels, patients exhibited significantly smaller LVEDVi (*P* = 0.04), LVSVi (*P* = 0.04), RVEDVi (*P* = 0.04) and RVSVi (*P* = 0.04), whereas left ventricular concentricity (*P* = 0.04) and septal thickness (*P* < 0.001) were both significantly larger. As smoking and physical inactivity may co-occur, we repeated the analyses covarying for both smoking and physical activity levels. The results remained essentially the same as the physical activity-adjusted analyses (supplementary Table 1). Adjusting for scanner (1.5 *v.* 3 T) did not affect the significance of any of the results (supplementary Table 3).

#### Associations between cardiac measures, total medication dose and duration of treatment

In linear regression analyses, no cardiac measure significantly correlated with total current chlorpromazine dose equivalents (supplementary Table 4) or with total chlorpromazine equivalents/years (supplementary Table 5).

## Discussion

In this study we found that people with schizophrenia show clinically significant reductions in indexed end-diastolic, end-systolic and stroke volumes in both ventricles (with large effect sizes), while showing an increase in concentricity and septal thickness (with large to very large effect sizes), compared with healthy controls of a similar age, gender, ethnicity, BSA and blood pressure. Mean concentricity in patients was above the reference range, whereas other measures were significantly different compared with controls, but mean values were within reference ranges. These findings were unchanged in sensitivity analyses adjusting for smoking or exercise levels, other than biventricular end-systolic volumes, which were no longer significantly different. In other words, for the first time to our knowledge, this study shows evidence consistent with concentric remodelling of the heart in schizophrenia. Cardiac remodelling is caused by heart myocytes' response to noxious stimuli, leading to structural changes, a process that can become pathogenic. Specifically, concentric remodelling is defined as that happening when volumes are normal or reduced, but the left ventricular mass-to-volume ratio is increased. Furthermore, the changes found in our sample of individuals with schizophrenia were independent of conventional cardiometabolic risk factors such as differences in age, gender, ethnicity, medical comorbidity, BSA, HbA_1c_ levels or blood pressure – something that previous studies^10–12^ could not consistently exclude and that could therefore account for the difference in results.

### Implications of our findings

Concentric cardiac remodelling has been shown to be among the best CMR predictors of future myocardial infarction, coronary insufficiency, heart failure and stroke in healthy adults: individuals in the highest tertile of concentricity showed the lowest cardiovascular disease-free survival over time and steepest slope curves among other common CMR measures, with a hazard ratio for CVD of 1.40 per 0.2 g/mL increase in concentricity.^26^ In a separate study, Bluemke and colleagues found a hazard ratio for coronary heart disease of 2.1 per g/mL and for stroke of 4.2 per g/mL increase in cardiac concentricity.^27^ Concentric heart remodelling was also shown to be a predictor of higher mortality (with a relative risk for all-cause mortality in individuals with concentric remodelling of 1.99) even in people with normal ejection fractions, such as our sample.^28^ Finally, increased left ventricular septal thickness, which is increased in our sample, also confers higher cardiovascular morbidity and mortality.^29^ The cardiac changes we report could therefore account for part of the increased cardiovascular morbidity and mortality seen in schizophrenia, independent of the increased prevalence of conventional cardiovascular risk factors, such as smoking, associated with schizophrenia.

### Interpretation of our findings

#### Potential causes of cardiac remodelling in schizophrenia

The most common cause of concentric heart remodelling in the general population are hypertensive changes, characterised by arterial stiffening causing elevated blood pressure.^30^ However, we found that in patients with schizophrenia concentric remodelling was present in the absence of signs of clinical or preclinical vascular disease, as demonstrated by blood pressure and pulse-wave velocity, which were both within normal range in patients, and not significantly different from controls. Our findings therefore suggest that concentric heart remodelling in schizophrenia might be a consequence of a separate pathophysiological process.

Until now, it was thought that most of the additional cardiometabolic risk in schizophrenia was due to unhealthy lifestyles and antipsychotic medication. However, a systematic review of potential aetiologies would include both genetic and environmental causes. First, genetic elements might exist that increase the risk of both schizophrenia and cardiac remodelling (either directly, or via mediators such as inflammatory cytokines). Second, environmental factors might also play a role. Prenatal and early life stressors have been linked to increased risk of both schizophrenia^31^ and cardiomyopathy and cardiac adverse outcomes^32^ later in life; these associations might be mediated by inflammation and other factors. Additionally, an unhealthy adult lifestyle is an established factor in increasing cardiac risk;^33^ individuals with schizophrenia have a tendency to lead a more unhealthy lifestyle than the general population (being more sedentary,^34^ smoking more,^35^ showing a preference for unhealthy foods^35^). Finally, people with schizophrenia take antipsychotic medication, a medication class, especially second-generation antipsychotics, that has been associated with metabolic syndrome^36^ and other cardiovascular risk factors.

#### Antipsychotic medication as a potential source of heart remodelling

With regard to antipsychotic treatment, there is animal evidence that D_2_ dopamine receptor signalling contributes to regulating cardiac function.^37^ There is also evidence that antipsychotics impair cardioprotection by blocking D_2_ receptors in a rat model of hypoxia-induced cardiomyocyte injury,^37^ thus potentially contributing to cardiac fibrosis. In rats, antipsychotics are also associated with the development of inflammatory lesions and lymphocytic infiltrates within the myocardium.^38^ In humans, it remains to be determined whether antipsychotics directly act on cardiomyocytes. Aside from this, some, but not all, antipsychotics have been associated with higher rates of metabolic syndrome,^36^ which might mediate cardiovascular changes. Therefore, antipsychotic treatment could account for some of the cardiac changes we demonstrate. However, in our sample we did not find a linear relationship between cardiac changes and current or lifetime antipsychotic dose. The absence of a linear relationship reduces the probability that a causal relationship exists,^39^ even if it cannot be ruled out. Additionally, a proportion of our study participants were taking clozapine, a medication that in rare, idiosyncratic occurrences can cause clozapine-induced myocarditis (CIM)^40^ and cardiomyopathy (CIC); the estimated combined prevalence of CIM and CIC in clozapine-treated patients is 0.93% (World Health Organization data in Bellissima *et al*^41^). Furthermore, the median time to onset of myocarditis since starting clozapine is 15 days,^40^ and we excluded participants with a history of myocarditis, cardiomyopathy or other cardiac disease. However, could participants with subclinical CIM/CIC be driving our findings? This is unlikely to have a major effect, as (a) less than 1% of clozapine-treated patients experience CIM or CIC so, even if the prevalence of subclinical changes is fourfold higher than the prevalence of clinical CIM/CIC, we would expect it to affect at most one patient in our sample; and (b) clozapine-induced myocarditis/cardiomyopathy generally shows an acute presentation over a few days, so it is unlikely that we have scanned patients with emergent CIM/CIC. Moreover, the major long-term sequela of CIM is dilated cardiomyopathy with chronic heart failure,^42^ and our findings point in the opposite direction: we find no evidence of heart failure (no change in ejection fractions) and evidence of concentric, rather than dilatatory, changes in our study population. Notwithstanding this, future studies will be useful to clarify the role of clozapine and other antipsychotic medication in the cardiac remodelling we find in schizophrenia.

#### Pathophysiological mechanisms potentially involved in cardiac remodelling in schizophrenia

We previously found evidence suggesting that an early diffuse myocardial fibrosis and/or subclinical myocardial inflammation are present in the hearts of people with schizophrenia.^43^ This would suggest that concentric heart remodelling and cardiac fibro-inflammatory changes could both be driven by systemic inflammation, leading to pro-fibrotic stimuli and hence cardiac remodelling with deposition of collagen. In support of this theory, there are established cross-sectional associations between schizophrenia and elevations in peripheral pro-inflammatory cytokines, including interleukin-6 (IL-6) and transforming growth factor β (TGF-β).^44^ Furthermore, elevated plasma levels of inflammatory cytokines early in life increase the risk of the subsequent development of a psychotic illness,^45^ suggesting that inflammation and psychosis might share a common early developmental aetiology. In particular, TGF-β has been consistently found to be elevated in peripheral blood in the early phases of schizophrenia as compared with healthy controls in several meta-analyses.^44^ Finally, animal studies support an aetiological role for TGF-β in causing cardiac fibrosis: in mice, TGF-β_1_ has been shown to have cardiac pro-fibrotic properties *in vivo*; in a transgenic model, overexpression of TGF-β_1_ resulted in a 38% increase in heart weight, with fibroblast proliferation and collagen synthesis in the myocardium.^46^ Therefore, inflammation and elevations in specific pro-inflammatory and pro-fibrotic factors might be mediating cardiac remodelling in schizophrenia, but this will need testing specifically in future studies.

### Strengths and limitations

A strength of this study is the use of CMR, which is the gold standard for left ventricular function and mass quantification.^14^ Key advantages of CMR over echocardiography are (a) that ventricles can be imaged in their entirety and no geometrical assumptions need to be made in order to derive global whole-organ data;^13^ and (b) that we were able to perform image analysis masked to participant diagnosis.

Another strength of our study is that, for the first time to our knowledge, participants with a pre-existing diagnosis of any medical condition, including ischaemic heart disease, hypertension, diabetes or dyslipidaemia, were excluded, and we matched patients and controls for BSA, age, gender and ethnicity, all potential confounders in analyses of cardiac function and structure.^14,47^ However, although we excluded diagnosed cardiometabolic disorders, we cannot exclude the possibility of subclinical changes, although we found no significant difference between patients and controls in systolic and diastolic blood pressure or in pulse-wave velocity, and no significant difference in HbA_1c_ in a subsample of our cohort, suggesting that subclinical differences are unlikely to be large.

A limitation of this study is that it involved people with chronic schizophrenia (median duration of treatment of 12 years), who were all taking antipsychotic medications. In addition, 70% of the patient group were taking clozapine and are therefore likely to be treatment resistant, which could affect the generalisability of our findings. We were not powered to detect differences in cardiac structure and function between antipsychotic treatments, and it would be useful for a future study to do this. Thus, a future study in antipsychotic-naïve patients would be useful to determine whether the cardiac differences we detect are associated with treatment or chronicity. Another consideration is that participants were scanned on two different scanners. However, findings remained significant when scanner was included in the analysis. Moreover, recent evidence shows that 1.5 T and 3 T scanners yield similar results for measuring cardiac structure and function,^48^ suggesting that the change in equipment is unlikely to have had a major effects on our results.

### Future directions

We observed biventricular volume reductions and evidence of concentric cardiac remodelling in patients with schizophrenia compared with matched healthy controls in the absence of concurrent established metabolic or medical risk factors. These are prognostically adverse changes and, in the absence of concurrent conventional metabolic or medical risk factors, could explain part of the additional cardiovascular morbidity and mortality seen in schizophrenia. Further work involving untreated participants is required to disentangle the contribution of antipsychotic medication to cardiac changes in schizophrenia.

## Data Availability

The dataset for this paper is available on Code Ocean, together with the R analysis codes, so that the analyses can be reproduced (https://doi.org/10.24433/CO.9265392.v1).

## Supplementary material

For supplementary material accompanying this paper visit https://doi.org/10.1192/bjp.2019.268. click here to view supplementary material
