# Supplementary material for: Cardiac structure and function in schizophrenia: cardiac magnetic resonance imaging study
Source: Br J Psychiatry. 2020 Aug;217(2):450–7. doi: 10.1192/bjp.2019.268 (PMC7511899; doi:10.1192/bjp.2019.268)
Supplement: Supplementary file 1 [file S000712501900268Xsup001.docx]

# Osimo et al; Cardiac structure and function in schizophrenia: a cardiac MR imaging study

**SUPPLEMENTARY INFORMATION**

## Supplementary figures

**Supplementary Figure 1: Cardiac Measurements in Patients with Schizophrenia and Healthy Controls.**

The graph shows cardiac measurements in patients with schizophrenia and healthy controls. There were no significant differences in left and right ventricle ejection fractions (LVEF p=0.94, RVEF p=0.97), in indexed left ventricular mass (LVMi, p=0.75), or in pulse wave velocity (PWV, p=0.59).
The points are individual patients’ values. In the box and whisker plot, the solid horizontal line is the median, the lower and upper hinges correspond to the first and third quartiles (the 25th and 75th percentiles), and the whiskers extends from the hinge to the largest/smallest value no further than 1.5*inter-quartile range from the hinge.

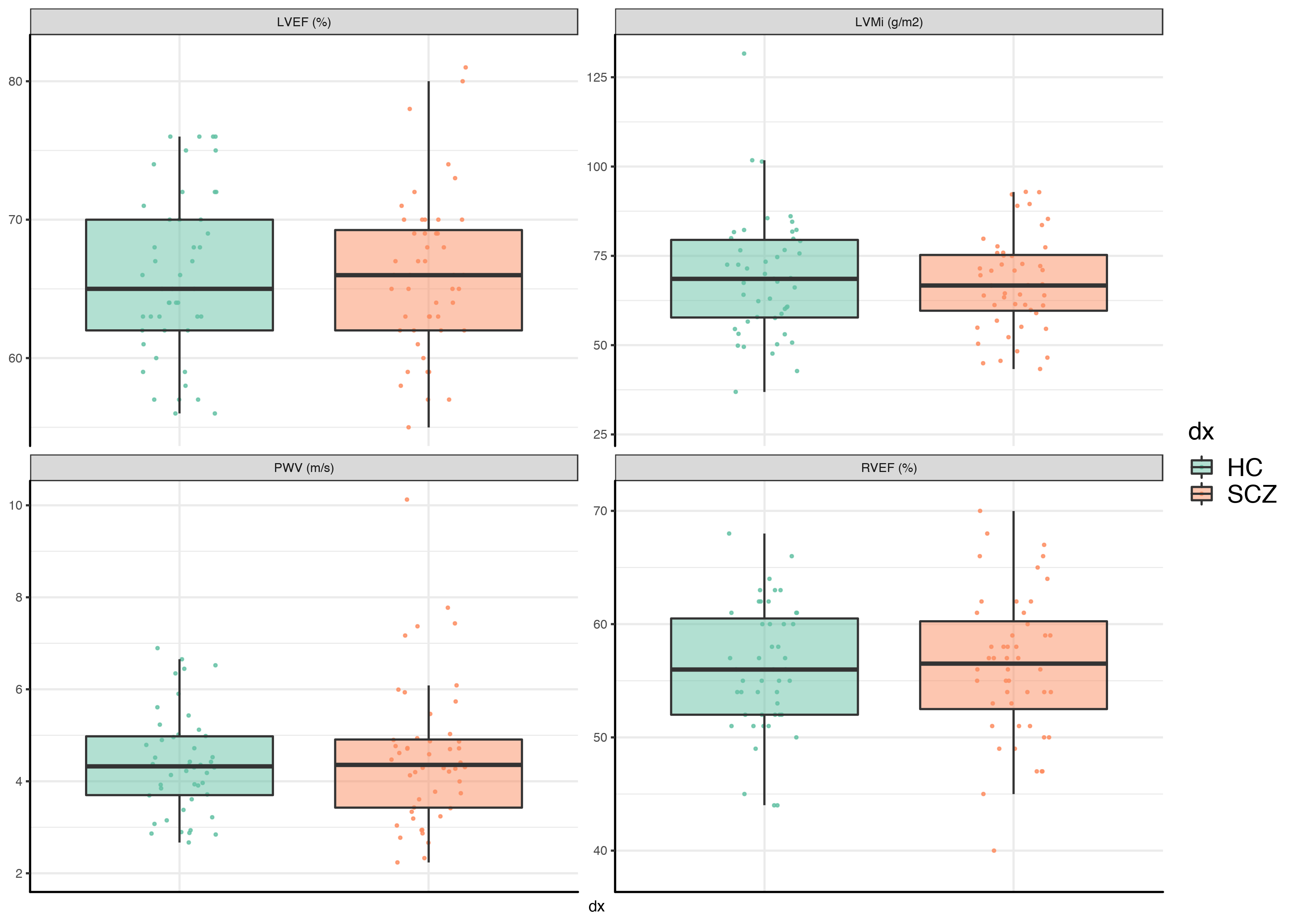


## Supplementary Tables

### Supplementary Table 1: CMR-Derived Cardiac Measurements in Patients with Schizophrenia and Matched Healthy Controls

Subjects in the two groups were matched for age, sex, ethnicity, BMI.

LVEDVi: indexed LV end-diastolic volume; LVESVi: indexed LV end-systolic volume; LVSVi: indexed LV stroke volume; LVMi: indexed LV mass; RVEDVi: indexed RV end-diastolic volume; RVESVi: indexed RV end-systolic volume; RVSVi: indexed RV stroke volume; EF: ejection fraction; PWV: pulse-wave velocity; BSA: body surface area; BH: Benjamini & Hochberg; SD: standard deviation; CI: confidence interval

|  |  |  |  | **Un-adjusted analysis** | | | **Adjusted for smoking (N of cigarettes)** | | **Adjusted for activity levels** | | **Adjusted for both smoking and activity levels** | |
| --- | --- | --- | --- | --- | --- | --- | --- | --- | --- | --- | --- | --- |
|  | **Schizophrenia**  **N=40**  **Mean (SD)** | **HCs**  **N=39**  **Mean (SD)** | **Normal range of parameters in males < 60 years (1)** | ***Schizophrenia***  ***F* (1, 77), p** | **BH-adjusted p value** | **Effect size (Cohen’s *d*; 95% CI)** | ***Schizophrenia F* (2, 75), p** | **BH-adjusted p value** | ***Schizophrenia F* (2, 75), p** | **BH-adjusted p value** | ***Schizophrenia F* (2, 75), p** | **BH-adjusted p value** |
| **LVEDVi (ml/m2)** | 68.48 (10.95) | 80.38 (17.40) | 64, 100 | **-11.90, 0.0005** | **0.001** | **-0.82;**  **-0.35--1.29** | **-11.68, 0.001** | **0.004** | **-8.67, 0.015** | **0.04** | **-8.47, 0.024** | **0.048** |
| **LVESVi (ml/m2)** | 23.45 (6.13) | 28.10 (9.67) | 17, 39 | **-4.65, 0.01** | **0.02** | **-0.58;**  **-0.12--1.03** | **-4.42, 0.025** | **0.04** | -3.02, 0.12 | 0.19 | -2.80, 0.177 | 0.18 |
| **LVSVi (ml/m2)** | 45.10 (7.38) | 52.35 (9.49) | 43, 67 | **-7.25, 0.0003** | **0.001** | **-0.85;**  **-0.39--1.32** | **-7.24,** **0.001** | **0.003** | **-5.64, 0.01** | **0.04** | **-5.64, 0.011** | **0.04** |
| **LVMi (g/m2)** | 68.44 (12.61) | 70.11 (17.23) | 57, 91 | -1.67, 0.62 | 0.75 | -0.11;  -0.34-0.56 | -2.5, 0.50 | 0.65 | -0.04, 0.99 | 0.99 |  |  |
| **RVEDVi (ml/m2)** | 80.88 (12.60) | 96.21 (24.68) | 63, 111 | **-15.32, 0.0008** | **0.002** | **-0.79;**  **-0.32--1.25** | **-14.79, 0.002** | **0.006** | **-10.89, 0.02** | **0.04** | **-10.38, 0.038** | 0.06 |
| **RVESVi (ml/m2)** | 35.84 (8.80) | 43.20 (15.65) | 18, 46 | **-7.36, 0.01** | **0.02** | **-0.58;**  **-0.12--1.04** | **-7.10, 0.02** | **0.04** | -5.24, 0.09 | 0.16 | -4.99, 0.13 | 0.15 |
| **RVSVi (ml/m2)** | 45.01 (7.54) | 53.10 (10.87) | 39, 71 | **-8.09, 0.0002** | **0.001** | **-0.87;**  **-0.40--1.33** | **-7.79, 0.0008** | **0.003** | **-5.80, 0.01** | **0.04** | **-5.52, 0.021** | **0.048** |
| **LV EF (%)** | 66.08 (5.71) | 65.85 (6.04) | 57, 75 | 0.23, 0.86 | 0.94 | 0.04;  -0.49-0.40 | -0.10, 0.94 | 0.99 | -0.37, 0.80 | 0.97 |  |  |
| **RV EF (%)** | 55.90 (6.50) | 55.95 (5.76) | 50, 78 | -0.05, 0.97 | 0.97 | -0.01;  -0.44-0.46 | -0.02, 0.99 | 0.99 | -0.08, 0.96 | 0.99 |  |  |
| **LV concentricity (g/ml)** | **1.01 (0.17)** | 0.88 (0.17) | **<0.91** | **0.13, 0.002** | **0.003** | **0.73;**  **0.27-1.19** | **0.10, 0.01** | **0.02** | **0.11, 0.01** | **0.04** | **0.09, 0.043** | 0.06 |
| **Septal thickness (mm)** | 10.49 (1.62) | 8.79 (1.37) | <15mm | **1.71, 0.000003** | **0.00004** | **1.13;**  **0.65-1.62;** | **1.79, 0.000004** | **0.00004** | **1.71, 0.00002** | **0.0002** | **1.81, 0.000016** | **0.0001** |
| **PWV (m/s)** | 4.75 (1.58) | 4.52 (1.03) | N/A | 0.23, 0.44 | 0.59 | 0.18;  -0.62-0.28 | 0.13, 0.68 | 0.82 | 0.08, 0.81 | 0.97 |  |  |

### Supplementary Table 2: CMR-Derived Cardiac Measurements in Patients with Schizophrenia and Matched Healthy Controls – Results adjusted for smoking as a binary factor

Subjects in the two groups were matched for age, sex, ethnicity, BMI.

BH: Benjamini & Hochberg

|  | **Adjusted for scanner difference** | |
| --- | --- | --- |
|  | ***Schizophrenia F* (2, 78), p** | **BH-adjusted p value** |
| **Indexed LV end-diastolic volume** | **-12.92, 0.0008** | **0.0025** |
| **Indexed LV end-systolic volume** | **-5.03, 0.02** | **0.033** |
| **Indexed LV stroke volume** | **-7.89, 0.0005** | **0.0025** |
| **Indexed LV mass** | -4.20, 0.27 | 0.36 |
| **Indexed RV end-diastolic volume** | **-15.15, 0.003** | **0.008** |
| **Indexed RV end-systolic volume** | **-7.02, 0.03** | **0.048** |
| **Indexed RV stroke volume** | **-8.27, 0.0008** | **0.002** |
| **LV ejection fraction** | 0.012, 0.99 | 0.99 |
| **RV ejection fraction** | -0.40, 0.80 | 0.87 |
| **LV concentricity (LV mass / LVEDV)** | **0.09, 0.03** | **0.048** |
| **Septal thickness** | **1.48, 0.0002** | **0.002** |
| **Pulse wave velocity** | 0.14, 0.69 | 0.82 |

### Supplementary Table 3: CMR-Derived Cardiac Measurements in Patients with Schizophrenia and Matched Healthy Controls – Results adjusted for scanner

Subjects in the two groups were matched for age, sex, ethnicity, BMI.

BH: Benjamini & Hochberg

|  | **Adjusted for scanner difference** | |
| --- | --- | --- |
|  | ***Schizophrenia F* (2, 78), p** | **BH-adjusted p value** |
| **Indexed LV end-diastolic volume** | **-11.96, 0.0004** | **0.001** |
| **Indexed LV end-systolic volume** | **-4.68, 0.01** | **0.02** |
| **Indexed LV stroke volume** | **-7.29, 0.0002** | **0.001** |
| **Indexed LV mass** | -1.67, 0.63 | 0.75 |
| **Indexed RV end-diastolic volume** | **-15.34, 0.0008** | **0.002** |
| **Indexed RV end-systolic volume** | **-7.33, 0.01** | **0.02** |
| **Indexed RV stroke volume** | **-8.13, 0.0002** | **0.001** |
| **LV ejection fraction** | 0.23, 0.87 | 0.94 |
| **RV ejection fraction** | -0.09, 0.94 | 0.95 |
| **LV concentricity (LV mass / LVEDV)** | **0.12, 0.002** | **0.003** |
| **Septal thickness** | **1.70, 2.45e-06** | **0.00003** |
| **Pulse wave velocity** | 0.24, 0.44 | 0.59 |

### Supplementary Table 4: Results of the linear regression analysis of the relationship between CMR-Derived Cardiac Measurements and total chlorpromazine-equivalent dose

|  | **R^2^** | **F(1,37), p** | **BH-adjusted p value** |
| --- | --- | --- | --- |
| **Indexed LV end-diastolic volume** | 0.14 | 0.01, 0.02 | 0.18 |
| **Indexed LV end-systolic volume** | 0.12 | 0.006, 0.03 | 0.18 |
| **Indexed LV stroke volume** | 0.09 | 0.005, 0.06 | 0.21 |
| **Indexed LV mass** | 0.09 | 0.01, 0.07 | 0.21 |
| **Indexed RV end-diastolic volume** | 0.06 | 0.009, 0.12 | 0.29 |
| **Indexed RV end-systolic volume** | 0.04 | 0.005, 0.25 | 0.43 |
| **Indexed RV stroke volume** | 0.05 | 0.004, 0.19 | 0.38 |
| **LV ejection fraction** | 0.01 | -0.001, 0.46 | 0.61 |
| **RV ejection fraction** | <0.001 | <0.001, 0.94 | 0.99 |
| **LV concentricity (LV mass / LVEDV)** | 0.01 | <0.001, 0.85 | 0.99 |
| **Septal thickness** | 0.02 | <0.001, 0.37 | 0.56 |
| **Pulse wave velocity** | <0.001 | <0.001, 0.99 | 0.99 |

### Supplementary Table 5: Results of the linear regression analysis of the relationship between CMR-Derived Cardiac Measurements and the natural logarithm of total chlorpromazine-equivalents/years

|  | **R^2^** | **F(1,35), p** | **BH-adjusted p value** |
| --- | --- | --- | --- |
| **Indexed LV end-diastolic volume** | 0.001 | -0.21, 0.87 | 0.94 |
| **Indexed LV end-systolic volume** | 0.004 | -0.27, 0.71 | 0.94 |
| **Indexed LV stroke volume** | 0.0003 | 0.07, 0.92 | 0.94 |
| **Indexed LV mass** | 0.006 | 0.72, 0.63 | 0.94 |
| **Indexed RV end-diastolic volume** | 0.008 | -0.80, 0.60 | 0.94 |
| **Indexed RV end-systolic volume** | 0.013 | -0.75, 0.49 | 0.94 |
| **Indexed RV stroke volume** | 0.0001 | -0.06, 0.94 | 0.94 |
| **LV ejection fraction** | 0.01 | 0.46, 0.49 | 0.94 |
| **RV ejection fraction** | 0.02 | 0.65, 0.39 | 0.94 |
| **LV concentricity (LV mass / LVEDV)** | 0.008 | 0.01, 0.60 | 0.94 |
| **Septal thickness** | 0.02 | 0.18, 0.37 | 0.94 |
| **Pulse wave velocity** | 0.09 | 0.32, 0.08 | 0.92 |

## Supplementary References:

1. Kawel-Boehm N, Maceira A, Valsangiacomo-Buechel ER, Vogel-Claussen J, Turkbey EB, Williams R, et al. Normal values for cardiovascular magnetic resonance in adults and children. Journal of Cardiovascular Magnetic Resonance. 2015; 17(1): 29.
